# Artelon as a Bio-Scaffold to Augment Collateral Ligament Repair after Knee Dislocation

**DOI:** 10.5704/MOJ.2207.014

**Published:** 2022-07

**Authors:** DM Myers, S Hyland, A Paulini, A Melaragno, BJ Passias, BC Taylor

**Affiliations:** Department of Orthopaedic Surgery, OhioHealth, Columbus, United States

**Keywords:** multiligamentous knee disruption, knee dislocation, bioaugmentation, artelon, graft

## Abstract

**Introduction:**

Knee dislocations (KD) have high rates of multi-ligamentous injury (MLI). Collateral ligaments rupture in 50-60% of KDs. Traditionally, collateral ligaments have undergone primary repair, though microscopic healing is not optimal. Artelon is a degradable, polyurethane urea bio-scaffold thought to decrease mechanical forces and promote healing, motion, and strength. Currently, little evidence exists regarding its indications or outcomes.

**Material and methods:**

Thirty-two patients with KD and MLI undergoing collateral ligament repair at a level-I trauma centre between 2015-2020 were included. Patients age <18, with ipsilateral fractures or inadequate follow-up were excluded. The Artelon (AG) and primary ligamentous repair group (PR) each included 16 patients. Injury and perioperative variables were evaluated using SPSS® .

**Results:**

Thirty-two KDs were included in 32 patients, with 60% anterior. There were no significant differences between the two cohorts demographically or with regards to the type or severity of injury sustained. Meniscal pathology was addressed in 14 patients in both groups. Thirty-eight percent of all patients lacked >15° of knee flexion. Only one gross failure occurred, in the AG. No differences were noted in infection or re-operation. Lysholm Knee Scale and Tegner Activity Scale were not significantly different, although Tegner scores in both cohorts decreased from pre-injury scores.

**Conclusions:**

In summary, Artelon appears to be safe without increasing risk for hypersensitivity or infection when used for collateral ligament augmentation. Additionally, Artelon appeared to be non-inferior and statistically equivalent to primary repair in this setting and may have promise with use in certain types of knee dislocations.

## Introduction

Knee dislocations (KD) are relatively uncommon, accounting for approximately 0.2% of orthopaedic injuries1. However, when knee dislocations occur, they are severe, with rates of associated fracture and vascular injury around 40% and 15%, respectively^1-3^. Multi-ligamentous disruption often occurs with knee dislocations. Most commonly, both the anterior cruciate ligament (ACL) and posterior cruciate ligament (PCL) are torn; the lateral collateral ligament (LCL) and the medial collateral ligament (MCL) are involved in 50-60% of cases^4^. Schenck classified KD based on the ligamentous disruption present, with KDIII injuries indicating ruptures of the ACL, PCL, and a collateral ligament and KDIV representing ruptures of the ACL, PCL, MCL, and LCL^5^. KDIII and KDIV injuries represent the majority of knee dislocations^3,4^.

While no “gold standard,” exists in the management of KDs and concomitant multi-ligamentous disruption, most agree a combination of ligamentous repair and reconstruction provides optimal results compared to non-operative management^6-11^. Historically, this included the reconstruction of the cruciate ligaments and primary suture anchor repair of the collateral ligaments^10-12^. While primarily repaired collateral ligaments demonstrate quicker remodelling and less laxity than those treated non-operatively, microscopic healing is still not optimal1^3-15^. Ligamentous healing is a process of haemorrhage, inflammation, proliferation, and remodelling that tends to promote scar rather than ligamentous regeneration^15-16^. This scar formation likely correlates with smaller, flawed collagen fibrils and a lack of collagen organisation leading to increased ligamentous creep, decreased stiffness, and sub-optimal tensile strength compared to native collateral ligaments^14,17-23^. In addition to structural compromise, collateral ligament weakness is exaggerated when multi-ligamentous knee injury occurs^16,24,25^. For these reasons, recovery time is prolonged for KDs with multi-ligamentous disruptions and is correlated with high rates of painful arthrofibrosis^26^.

In response to suboptimal microscopic healing and the need for early range of motion (ROM) to avoid knee stiffness, interest has grown in bioaugmentation scaffolds. Looney *et al*^27^ evaluated current concepts in bio-scaffolds, stating that they should possess certain characteristics: a three-dimensional nature for cell growth and nutrient transport, biocompatibility with controllable degradation, suitable chemistry for cell attachment and proliferation, and mechanical properties matching those of the tissue being treated^27-29^. Several new bio-scaffold products have been introduced in the last decade with promising results. Smith *et al*^30^ looked at the use of a variety of commercial bio-scaffolds, including the synthetic bio-scaffolds Biofiber [Tornier, Bloomington, MN] and X-repair [Synthasome, Del Mar, CA], and found that they helped promote increased cell adhesion and tendon-like characteristics after repair of rotator cuffs. Proctor^31^ showed that the X-repair augmentation in massive rotator cuff repairs resulted in 78% of patients having substantial functional improvement at 42 months.

Regarding bio-scaffold augmentation in ligamentous repair, limited literature exists. Of the commercially available synthetic bio-scaffolds, Artelon [Artelon, Marietta, GA] offers promise for ligamentous use, and was chosen for this reason^32,33^. Made from a synthetic biodegradable polyurethane urea, Artelon is of particular interest given its strong, creep-resistant, inert properties that are similar to ligaments. Additionally, Artelon is not thought to cause hypersensitivity^29,34-36^. These principles, theoretically, allow Artelon to decrease forces on the ligament, while promoting host cell infiltration and biologic healing, acting as an internal brace of sorts. One animal study demonstrated Artelon’s efficacy in achieving faster ROM return and improved post-operative pain^37^. However, there are no known studies regarding Artelon collateral ligament augmentation after KD and MLI. Therefore, clinical outcomes after this type of augmentation are currently unknown. The primary objective of our study was to investigate the clinical outcomes and safety of collateral ligament augmentation using Artelon after KD, with the goal of promoting improved strength, early ROM, and decreased laxity.

## Materials and Methods

Following institutional review board approval and informed consent, a retrospective chart review was conducted on adult patients with multi-ligamentous knee injuries who underwent operative repair of collateral ligaments after sustaining a knee dislocation between January 1st, 2015, and July 31st, 2020. It was used in cases where it was thought it would provide improved collateral stability. Three fellowship-trained orthopaedic traumatologists (BT, SM, JC) contributed, all at an urban Level-1 Trauma Centre in the central United States. Collateral ligaments were either primarily repaired with suture or augmented with Artelon. Exclusion criteria included patients <18 years old, those with concomitant ipsilateral lower extremity fractures, and those with no documentation of physical examination findings at follow-up. After exclusion of six patients, a total of 32 patients were included in the study, with 16 patients making up the Artelon group (AG) and 16 patients forming the primary ligamentous repair group (PR). The series was consecutive with primary repair initially being performed, switching to Artelon augmentation as the study progressed.

Injury characteristics, including the mechanism and energy of the KD event, were recorded. In addition, of the 32 patients, 28 underwent detailed magnetic resonance imaging (MRI) analysis reviewing ACL, PCL, MCL, and PLC integrity. Also noted were concomitant intra-articular knee injuries not involving collateral or cruciate ligaments, for example, meniscal tears or osteochondral injuries. The four patients without MRI were taken emergently to the operating room for reduction of irreducible KDs. Ligamentous examination was performed under anaesthesia before all surgeries in both groups, regardless of pre-operative MRI. Examination included a Lachman test, posterior drawer test, and varus/valgus stress at 0/30° flexion to test all ligamentous integrity. Therefore, final ligamentous injury diagnosis and Schenck^5^ grade were a combination of intra-operative and MRI findings (Table I). LCL injuries were included as PLC injuries for the purpose of statistical analysis.

**Table I:** Injury data

Variable	Artelon Group (n=16)	Primary Repair Group (n=16)	P value
High Energy	13 (81%)	10 (63%)	0.43
Low Energy	3 (19%)	6 (38%)	0.43
Complete ACL Injury	11 (69%)	14 (88%)	0.39
Incomplete ACL Injury	5 (31%)	2 (12%)	0.39
Complete PCL Injury	7 (70%)	8 (62%)	1.0
Incomplete PCL Injury	3 (30%)	5 (38%)	0.69
Complete MCL Injury	4 (44%)	5 (38%)	1.0
Incomplete MCL Injury	5 (56%)	8 (62%)	0.47
Complete PLC Injury	9 (64%)	9 (75%)	1.0
Incomplete PLC Injury	5 (36%)	3 (25%)	0.69
Schenck KDIII	11 (69%)	7 (44%)	0.29
Schenck KDIV	5 (31%)	9 (56%)	0.29
Lateral meniscus tear	11 (69%)	9 (56%)	0.72
Medial meniscus tear	11 (69%)	6 (38%)	0.16
Osteochondral injury	3 (19%)	0 (0%)	0.23

* Values are expressed as means + standard deviations or as absolute values with percentages in parentheses

Intra-operatively, collateral ligament repair/augmentation was undertaken at the index surgery, followed by a period of the patient wearing a hinged-knee brace to promote ROM and capsular reconstitution. Typically, cruciate repair and concomitant injuries were addressed three-weeks later.

**Fig 1:**
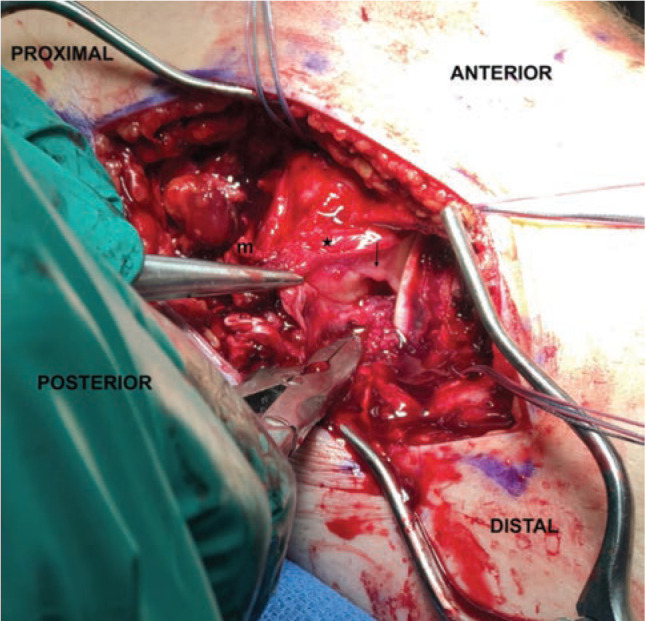
Traumatic MCL rupture (‘m’) to left knee with capsular (star) and meniscal disruption (arrow).

**Fig 2:**
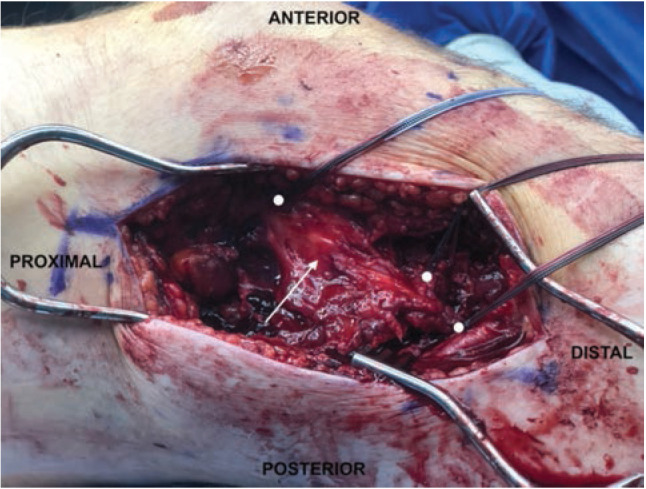
Primary repair of traumatic left knee MCL rupture (arrow) using femoral and tibial suture anchors which are signified by circles.

**Fig 3:**
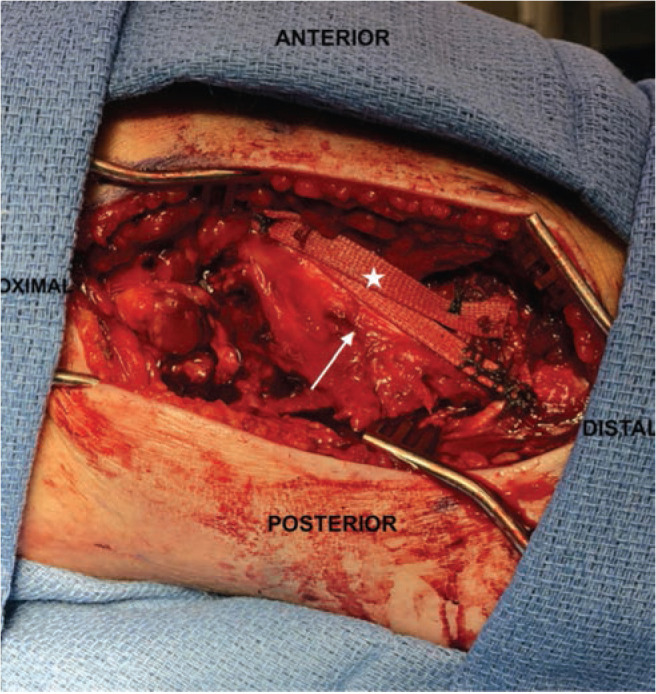
Primary left knee MCL repair (arrow) with overlying Artelon augmentation (star), fixed with suture anchors.

**Fig 4:**
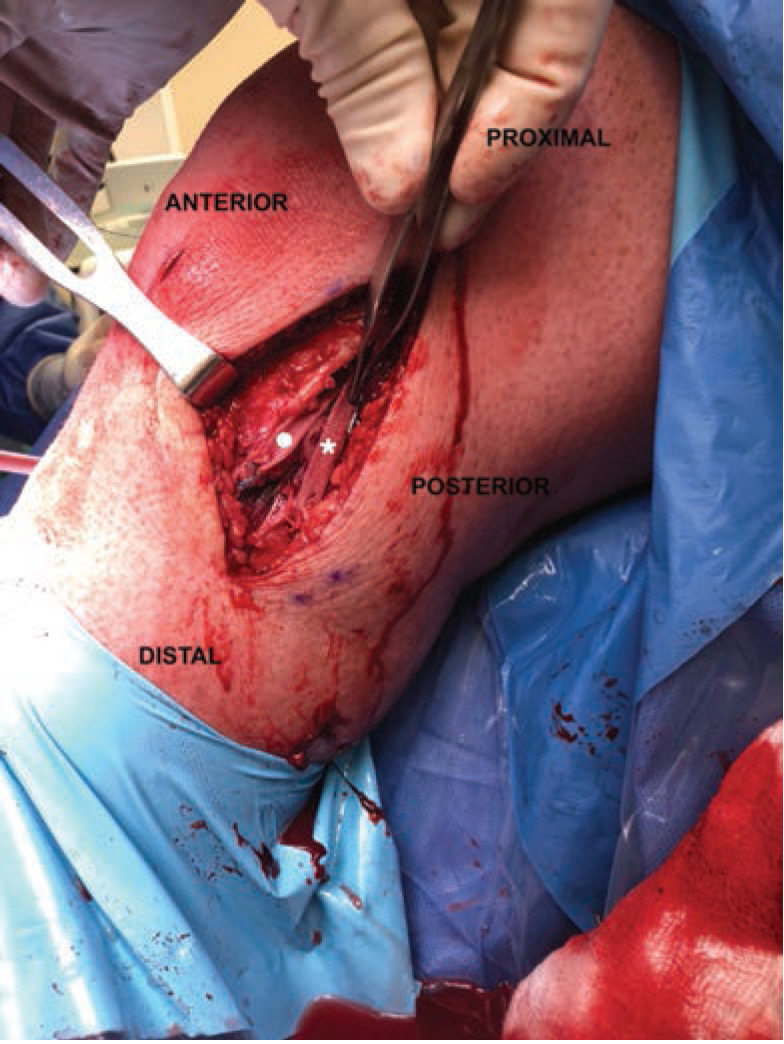
PLC repair of left knee with overlying Artelon augmentation into proximal fibular (star) and tibial (circle) suture anchors.

In reference to the index surgery, collateral ligaments were addressed with an open approach. Soft tissue dissection was performed medially or laterally for the MCL or PLC, respectively, being careful to avoid neurovascular structures at risk. Any capsular disruption (Fig. 1) deep to the ligamentous structure was repaired with absorbable figure-of-eight sutures. Once the torn ligament was evaluated (Fig. 2), the surgeon decided if Artelon augmentation would be included in the ligamentous repair. A primary ligamentous repair was first performed using suture anchors at isometric points on the distal femur proximally and distally at either the fibular head (PLC) or the proximal medial tibia approximately 5-6cm inferior to the joint line (MCL). This was done using a locked, running suture configuration. Next, an appropriate length 0.7mm thick Artelon graft was laid over top the collateral ligament repair and fixed isometrically with suture from the anchors proximally and distally and incorporation of the underlying collateral repair (Fig. 3). In the case of PLC augmentation with Artelon, an additional suture anchor was placed anterior to the fibular head anchor into the proximal tibia and sewn into the Artelon graft to provide improved rotational stability (Fig. 4). In the PR group, similar steps were taken regarding suture anchor fixation for ligamentous disruption, but no Artelon augmentation was employed.

Three weeks after collateral stabilisation, cruciate ligaments were addressed arthroscopically. ACL reconstruction was performed in 28 patients using a tibialis anterior allograft to perform an all-inside reconstruction with Arthrex TightRope [Arthrex, Inc; Naples, FL] configuration. No PCL reconstructions or repairs were undertaken in either study cohort.

Operative variables were recorded for each patient, including ligaments repaired or reconstructed during surgery and whether Artelon was used for augmentation (Table II). In two cases, Artelon was used to augment both the MCL and PLC. Patients undergoing associated meniscectomy, meniscus repair, or chondroplasty due to aforementioned intra-articular pathology were documented because of the high association with these injuries^38^.

**Table II:** Operative variables

Variable	Artelon Group (n=16)	Primary Repair Group (n=16)	P value
Length of Surgery (mins)	102 + 29.5	132 + 45.4	0.03
ACL Reconstruction	15	13	0.60
PCL Reconstruction	0	0	1.0
MCL Repair	8	10	0.72
PLC Repair	10	11	1.0
Concomitant meniscectomy	12	12	1.0
Concomitant meniscal repair	2	2	1.0
Concomitant chondroplasty	9	6	0.48

* Values are expressed as means + standard deviations or as absolute values with percentages in parentheses

Patient follow-up information was tracked and recorded during the patient’s recovery. Average follow-up time was 41 weeks in the PR group and 36 weeks in the AG. Time to weight-bearing was recorded, with most patients being permitted to weight-bear as tolerated in a hinged-knee brace locked in full extension post-operatively. ROM was restricted to 0-30° initially, given the severity of these injuries. Formal outpatient physical therapy was started, at the latest, two weeks after final surgery. The laxity grade^38^ of involved ligaments, the degree of continued subjective instability, and the knee ROM were documented at final post-operative visits. The degree of stiffness was quantified as mild (lacking <15°), moderate (lacking 15-30°), or severe (lacking >30°) for flexion and extension. Post-operative ligamentous failure or subsequent operations were recorded. Complications such as hypersensitivity reaction were also detailed. Additionally, patient outcome scores were calculated using Lysholm Knee Scale and Tegner Activity Scales pre- and post-injury/surgery (Table III). All data was collected using electronic medical records and stored in a secure database [Microsoft Excel].

**Table III:** Post-operative variables

Variable	Artelon Group (n=16)	Primary Repair Group (n=16)	P value
ACL Laxity (Grade 1)	15 (94%)	15 (94%)	1.0
PCL Laxity (Grade 1)	15 (94%)	16 (100%)	1.0
MCL Laxity (Grade 1)	15 (94%)	15 (94%)	1.0
PLC Laxity (Grade 1)	14 (88%)	16 (100%)	0.48
Subjective Valgus Instability (Y)	1 (6%)	1 (6%)	1.0
Subjective Varus Instability (Y)	2 (13%)	0 (0%)	0.48
Grade 1 (0-15) Knee Flexion Deficit	10 (63%)	10 (63%)	1.0
Grade 2 (15-30) Knee Flexion Deficit	3 (19%)	2 (13%)	1.0
Grade 3 (>30) Knee Flexion Deficit	3 (19%)	4 (25%)	1.0
Grade 1 (0-15) Knee Extension Deficit	16 (100%)	16 (100%)	1.0
Grade 2 (15-30) Knee Extension Deficit	0 (0%)	0 (0%)	1.0
Grade 3 (>30) Knee Extension Deficit	0 (0%)	0 (0%)	1.0
Ligamentous Failure (Y)	1 (6%)	0 (0%)	1.0
Infection (Y)	2 (13%)	0 (0%)	0.48
Hypersensitivity Reaction (Y)	0 (0%)	0 (0%)	1.0
Repeat Surgery (Y)	5 (31%)	4 (25%)	1.0
I&D (Y)	2 (13%)	0 (0%)	0.48
Knee Manipulation (Y)	0 (0%)	4 (100%)	0.10
Lysholm Knee Scale	63.1 + 20.5	69.3 + 20.8	0.34
Tegner Activity Score Pre-Injury	4.8 + 1.4	5 + 1.0	0.75
Tegner Activity Score Post-Injury	2.9 + 2.4	3.6 + 1.8	0.34
Decrease in Tegner Score Pre/Post Injury	1.9 + 1.4	1.22 + 1.4	0.30

* Values are expressed as means + standard deviations or as absolute values with percentages in parentheses

After data collection, statistics were analysed using SPSS® with means, ranges, and confidence intervals calculated for continuous variables and compared using Student’s t-tests. Frequencies were calculated for dichotomous variables and compared using Fisher’s exact test for increased accuracy in small proportion analysis. A significance level of p < 0.05 was set prior to investigation.

## Results

Overall, 32 patients were included in the study, 16 in the AG and 16 in the PR group. There were no statistically significant demographic differences between the groups (Table IV). The two groups were also similar with regards to the types of injuries studied and their severity, based on the Schenck classification (Table I). High energy mechanisms were the main cause of injury in both groups which consisted of motor vehicle collisions, pedestrian vs motorised vehicle, or a fall from height. In contrast, low energy mechanisms were defined as ground level falls or low direct impact injuries. Concomitant meniscal or osteochondral pathology were common but equal, noted in 15 patients in the AG group and 14 in the PR group. Intra-operatively, cruciate ligament reconstruction was consistent between groups in number, graft type, and technique. MCL repair was performed in 18 total patients, with eight undergoing Artelon augmentation. PLC repair, including LCL repair, was performed in 21 subjects, with 10 having undergone Artelon augmentation. Meniscectomy and meniscal repair rates were the same in both groups.

**Table IV:** Demographic data

Variable	Artelon Group (n=16)	Primary Repair Group (n=16)	P value
Age (Yrs)	42 + 14	42 + 13.9	0.92
Gender (Male)	12 (75%)	12 (75%)	1.0
BMI (kg/m2)	36 + 13	30 + 11.5	0.25
Diabetic (Y)	1 (6%)	1 (6%)	1.0
Smoker (Y)	6 (38%)	4 (25%)	0.70

* Values are expressed as means + standard deviations or as absolute values with percentages in parentheses

Post-operatively, average length of follow-up was 36 weeks in the AG and 41 weeks in the PR group. One patient in each group was noted to have grade two/three MCL laxity on examination, while two patients in the AG were noted to have increased laxity of the LCL. These patients also complained of subjective instability with valgus (MCL) and varus (LCL) stress. Post-operative stiffness was common in both groups, particularly with a lack of flexion. Both cohorts had six patients with moderate/severe knee flexion limitations at final follow-up.

Gross ligamentous failure was uncommon in both groups, only occurring in one PLC repair in the AG. Two infections were noted, both in the AG (p=0.48) No abnormal hypersensitivity or soft tissue reactions were noted in either group and the need for repeat surgery was similar amongst the study cohorts, with the caveat that the PR group required four knee manipulations under anaesthesia. Lysholm Knee Scale for the AG was 63.1, compared to 69.3 for the PR group (p=0.30). Tegner pre-injury scores were 4.8 and 5.0 for the AG and PR groups, respectively (p=0.75). Both groups saw Tegner scores decrease after injury with AG scoring 2.9 and PR group 3.6 (p=0.34). Post-hoc power analyses for outcome scores demonstrated that the study may have been underpowered to represent significant differences.

## Discussion

Knee dislocations are significant orthopaedic events, which can have severe complications^1^. They are associated with multi-ligamentous knee dislocations, most commonly of the KDIII and KDIV variety^2-5^. In multi-ligamentous knee disruptions, cruciate ligaments are typically reconstructed, and collateral ligaments are variably repaired. Studies have suggested that, compared to non-operative management, primary repair of collateral ligament ruptures may decrease creep and laxity as well as improving load to failure and ultimate stiffness^23^. That being said, primary ligamentous healing still leads to increased laxity compared to native collateral ligaments due to flawed, disorganised collagen cross-linking, and scar tissue formation^14,17-21^. This loss in tensile strength has been recognised as long as 2 ½ years following primary repair^15^.

For these reasons, bio-augmentation has been investigated as a way to decrease forces on ligament repair, enhance healing, and allow for cell infiltration^29,32,36^. Artelon, specifically, is a polyurethane urea polymer synthetic scaffold that is partially degradable. It is thought that 50% of the scaffold remains at six years, which provides creep resistance and mechanical support, while allowing host integration over time^29,34,35^. Petranto *et al*^34^ described the favourable characteristics of Artelon and noted their successful use of Artelon in cases of repair of the Achilles, posterior tibialis, and peroneal tendons, where all patients returned to preinjury levels without limitation. The favourable mechanical properties, in theory, allow for earlier improved ROM, decreased long-term ligamentous failures and improved patient outcome scores.

This said, there is an overall scarcity of clinical studies published on Artelon use in humans. Shoaib *et al*^33^, used Artelon to augment chronic Achilles ruptures with large defects and noted no re-ruptures or infections in seven patients. Our study demonstrates similar clinical improvement in both cohorts, with Artelon non-inferior. This holds true for both MCL and PLC augmentation. In our study there was one episode of gross ligamentous failure and four patients who described subjective laxity. Four of the five episodes were noted in the AG cohort, not reaching statistical significance. The one patient with gross ligamentous failure opted not to have further surgery as he was still able to perform his daily activities in a brace. Generally, residual laxity was low in both groups, although increased patient volume may have altered these findings.

Gersoff *et al*^37^, used Artelon to augment eight patellar tendon ruptures in dogs and noted earlier function and ROM with improved outcomes. There are no known studies describing post-operative stiffness results or objective outcome measures using Artelon augmentation in humans. In the current study, post-operative arthrofibrosis was quite common in both groups, with 38% of both the AG and PR groups being noted to have mild/moderate knee flexion deficits. Lysholm and Tegner outcome scores were also not significantly different between the two groups. Interestingly, outcome scores were higher in the PR group pre-injury and post-operatively which may reflect pre-injury functional difference between groups.

Additionally, there have also been concerns linking Artelon to hypersensitivity reactions or an increased rate of infection. Bio-scaffold products in general have been questioned regarding their human compatibility, with foreign body rejection and inflammatory response as possible side effects^28,39,40^. The ability of the scaffold to degrade within a reasonable time-period is thought to decrease the amount of cellular inflammation^40^. It was thought that Artelon’s design would allow it to degrade enough to minimise hypersensitivity reactions but also continue to provide enough stability and cellular infiltration to allow for strong, ligamentous healing^29,32,34^. That being said, Robinson *et al*^41^ reported three cases of persistent pain after using Artelon to augment CMC joint arthroplasty and questioned the biocompatibility and incorporation of the scaffold. Our study, however, did not show any obvious hypersensitivity reactions. Two infections were noted, both in the AG cohort, not significantly more than the PR group. One of the patients with infection had a BMI of 57 and was also a smoker, but no other obvious risk factors were noted. Additionally, the failures noted in the aforementioned Robinson *et al*^41^ study all occurred within nine months. Theoretically, given this timeline, failures due to hypersensitivity could have been reasonably identified over our study course.

Additionally, the number of total concomitant intra-articular injuries was greater in the AG group^25^ compared to the PR group^15^, which may have ultimately influenced outcome scores and associated satisfaction. Despite this finding, outcome scores in the AG remained non-inferior. Unfortunately, it is difficult to isolate multiligamentous injuries after KD without other concomitant intra-articular pathology, given their commonality.

One of the primary limitations of this study is the small sample size, as demonstrated by post-hoc power analysis; however, given the relative rarity of knee dislocations and subsequent collateral ligament augmentation with Artelon, we feel our sample size is a reasonable representation of the problem in question. With a larger sample size, results may reach significance and may be more easily generalisable. Indications for Artelon use were also not well defined among the patients included in the study as this was decided by the surgeon at the time of repair. Given the retrospective nature of our study, the AG group also had, on average, five less weeks of follow-up than the PR group, as noted in the results. With equal follow-up times, outcome scores in the AG cohort as well as ROM values may have improved.

## Conclusion

Artelon use for collateral ligament augmentation after multi-ligamentous knee disruption was non-inferior to the PR group regarding post-operative knee stiffness or subjective/objective measures of instability. In addition, there does not appear to be an increased risk of hypersensitivity or infection with Artelon augmentation. At this time there is not enough sufficient evidence to formally recommend Artelon augmentation. Further investigation, such as studies that include a larger prospective cohort, would increase available data enabling investigators to define specific guidelines and appropriate clinical use recommendations to improve outcomes with Artelon augmentation.
